# Retrosplenial Cortex Contributes to Network Changes during Seizures in the GAERS Absence Epilepsy Rat Model

**DOI:** 10.1093/texcom/tgab023

**Published:** 2021-03-23

**Authors:** Lydia Wachsmuth, Maia Datunashvili, Katharina Kemper, Franziska Albers, Henriette Lambers, Annika Lüttjohann, Silke Kreitz, Thomas Budde, Cornelius Faber

**Affiliations:** Translational Research Imaging Center, Clinic for Radiology, University Hospital Münster, 48149 Münster, Germany; Institute of Physiology I, University of Münster, 48149 Münster, Germany; Translational Research Imaging Center, Clinic for Radiology, University Hospital Münster, 48149 Münster, Germany; Translational Research Imaging Center, Clinic for Radiology, University Hospital Münster, 48149 Münster, Germany; Translational Research Imaging Center, Clinic for Radiology, University Hospital Münster, 48149 Münster, Germany; Institute of Physiology I, University of Münster, 48149 Münster, Germany; Experimental and Clinical Pharmacology and Toxicology, University of Erlangen, 91054 Erlangen, Germany; Institute of Physiology I, University of Münster, 48149 Münster, Germany; Translational Research Imaging Center, Clinic for Radiology, University Hospital Münster, 48149 Münster, Germany

**Keywords:** epilepsy, fMRI, GAERS, graph theory, networks

## Abstract

Resting state-fMRI was performed to explore brain networks in Genetic Absence Epilepsy Rats from Strasbourg and in nonepileptic controls (NEC) during monitoring of the brain state by simultaneous optical Ca^2+^-recordings. Graph theoretical analysis allowed for the identification of acute and chronic network changes and revealed preserved small world topology before and after seizure onset. The most prominent acute change in network organization during seizures was the segregation of cortical regions from the remaining brain. Stronger connections between thalamic with limbic regions compared with preseizure state indicated network regularization during seizures. When comparing between strains, intrathalamic connections were prominent in NEC, on local level represented by higher thalamic strengths and hub scores. Subtle differences were observed for retrosplenial cortex (RS), forming more connections beyond cortex in epileptic rats, and showing a tendency to lateralization during seizures. A potential role of RS as hub between subcortical and cortical regions in epilepsy was supported by increased numbers of parvalbumin-positive (PV+) interneurons together with enhanced inhibitory synaptic activity and neuronal excitability in pyramidal neurons. By combining multimodal fMRI data, graph theoretical methods, and electrophysiological recordings, we identified the RS as promising target for modulation of seizure activity and/or comorbidities.

## Introduction

Absence epilepsy is a nonconvulsive form of epilepsy, accounting for 10–17% of all pediatric epilepsies (Crunelli and Leresche 2002). Although considered a mild form of epilepsy, absence seizures are associated with long-term behavioral changes and comorbidities. Impaired cognition, memory function, and attentional deficits have been reported (Crunelli et al. 2020).

Absence epilepsy is a network disease. The central element in the pathology and specifically in initiation, maintenance, and termination of the characteristic spike-and-wave discharges (SWDs) is the cortico-thalamo-cortical circuitry (Lüttjohann and Pape 2019). Combined electroencephalography (EEG) and functional (f)MRI studies (Gotman and Pittau 2011) confirmed involvement of similar brain network components in adult (Moeller et al. 2013; Carney and Jackson 2014) and pediatric patients (Aarabi et al. 2008; Berman et al. 2010). In addition to the widely accepted involvement of sensory cortex and thalamus, association cortex, limbic regions, and basal ganglia form brain circuities remote from this core network, which presumably influence seizure manifestation. Specifically, altered connectivities between retrosplenial cortex, anterior thalamus, and hippocampus were reported in the Pentylenetetrazol (PTZ) model of absence epilepsy and during experimental limbic seizures (Brevard et al. 2006; Englot et al. 2009). These changes were related to the loss of consciousness during seizures. The restrosplenial cortex, a brain region essential for several cognitive functions including learning and memory, is an important component of the so-called default mode network that is an important mediator of passive states of mind (Raichle et al. 2001; Chrastil 2018). It has been suggested (Carney and Jackson 2014) that this default mode network might permit or facilitate the occurrence of seizures in line with the observation that absence seizures in animal and man most often occur at times of fatigue and rest. Global brain network changes can be revealed by analysis of multisite electrical recordings and resting state-fMRI—rs-fMRI (as reviewed by Lehnertz et al. 2014). Functional connectivity in such studies is assessed by cross-correlating the respective temporal signal fluctuations across brain regions (Van Diessen et al. 2013). With graph theory (Rubinov and Sporns 2010), such correlation matrices are visualized and characterized quantitatively, where brain regions represent the nodes of the network. Correlation coefficients of regional signal time courses indicate the links between these nodes. Metrics define overall aspects of functional integration and segregation (small world topology), characterize community structure (modularity), and quantify importance of individual brain regions within the network. Literature concerning findings in epilepsy obtained by graph theory are not coherent. Increases (Horstmann et al. 2010) and decreases in clustering coefficient and in shortest path length (Zhang et al. 2011) or decreases in clustering coefficient accompanied by increased characteristic path length (Xiao et al. 2015) have been reported. Reasons for seemingly contradicting findings may be due to the specific subtype of epilepsy studied, the brain state recorded (pre-ictal, ictal, post-ictal, interictal) and the reference, results are compared to (intra-individually between brain states or interindividually to healthy controls). Evidence is accumulating for a shift toward network regularization during seizures (review by Kramer and Cash 2012).

In animal models of absence epilepsy, similar brain network components as in humans are involved (Nersesyan et al. 2004; Blumenfeld 2007; Mishra et al. 2011; Depaulis et al. 2016). Activation of the cortico-thalamic circuitry responsible for generating and maintaining SWDs has been confirmed by using fMRI (Tenney et al. 2003, 2004). When comparing global network parameters, studies in various rat models of epilepsy yielded mixed results. In a temporal lobe epilepsy (TLE) model, Christiaen et al. (2019) reported lower functional connectivity, a significantly lower clustering coefficient, and a higher characteristic path length compared with controls in the interictal state. In contrast, in the tetanus toxin rat model of focal epilepsy, an increased global clustering coefficient and characteristic path length were found (Otte et al. 2012). Gill et al. (2017) and also observed a significantly higher clustering coefficient and an increased small world index, but unchanged path length in the kainic acid model of TLE. Brain connectivity studies using rs-fMRI in absence epilepsy models are rare (Mishra et al. 2013; David et al. 2008) and none provided a general assessment of network topology. Mishra et al. found enhanced interhemispheric connectivity in cortical regions of Wistar Albino Glaxo rats from Rijswijk (WAG/Rij) rats, when comparing interictal versus ictal periods during data acquisition while animals experienced seizures, but not in runs without seizures. David et al. (2008) investigated the directionality of interactions between brain regions in Genetic Absence Epilepsy Rats from Strasbourg (GAERS) with Granger causality and dynamic causal modelling and found evidence for the role of the barrel field cortex as a driver of epileptic activity.

Here, we acquired rs-fMRI data from GAERS before and during seizures, and from NEC. GAERS (Depaulis et al. 2016) represent an established spontaneous rat model of absence epilepsy that displays many aspects of human pathology. Similar to humans, with sudden absences up to several hundred times per day, rats experience frequent seizures during 10–15% of their time in quiet wakefulness. Behavioral characteristics of an absence seizure in rat as in the human patient are transient impairment of consciousness, brief interruption of ongoing activity, and momentary unresponsiveness to the environment. Originally, no neurologic or neurocognitive deficits (Vergnes et al. 1991) were seen in GAERS. More recent studies in the Melbourne colony, indicated anxiety and depression-like behavior (Jones et al. 2008; Jones et al. 2010; Powell et al. 2014). Anatomical MRI in the same colony revealed increased cortex and amygdala volumes (Bouilleret et al. 2009). Learning and memory deficits have been observed more recently in GAERS (Marques-Carneiro et al. 2016) and in WAG/Rij rats (Karson et al. 2015).

We applied graph theoretical analysis of rs-fMRI data to characterize brain networks and to identify potential acute and chronic network changes in this absence epilepsy model.

## Methods

### Animals

Experiments were performed in line with ARRIVE guide lines with adult (>3 months), female GAERS (*n* = 25), and NEC (*n* = 20) from the breeding facility of the Institute of Physiology I, University of Münster (animal use protocols 84-02.04.2015.A427, 84-02.05.50.15.A026, Landesamt für Natur, Umwelt und Verbraucherschutz Nordrhein-Westfalen). Adult GAERS exhibit frequently occurring absence seizures with a prevalence of 100% (Depaulis et al. 2016). Rats were housed in cages of 2–4 animals at 21 °C, controlled relative humidity of 45–65%, a 12/12 h light/dark cycle with unlimited access to food and water. An overview of all animals is given in Supplementary Tables 1 and 2.

### Experimental Setup for In Vivo Experiments

**Figure 1:**
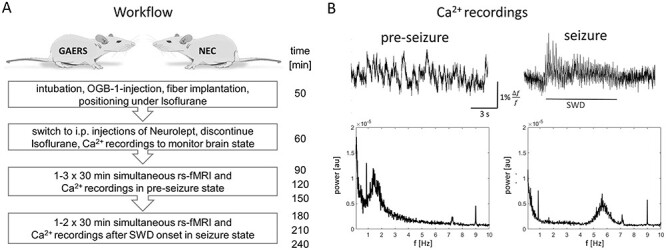
(*A*) Study workflow. Animal numbers are reported in Supplementary Table 1. (*B*) Optical Ca^2+^ recordings obtained after switching from Isoflurane to Neurolept. Upper row: exemplary 15 s Ca^2+^-traces show preseizure state (left) and seizure state (right). In seizure state, SWDs alternate with epochs of rest. Lower row: Power spectra of 2 exemplary 15 min Ca^2+^-traces of a GAERS rat: In preseizure state (left), mainly low-frequency fluctuations were observed. In seizure state (right), the typical frequency of SWDs at 6 Hz was detected.

The experimental workflow is displayed in Figure 1*A*. In order to monitor the brain state and the occurrence of the characteristic, generalized SWDs during seizures, we employed fiber-based, optical Ca^2+^ recordings (Albers et al. 2018). Animal preparation was performed under Isoflurane (Baxter vet.) anesthesia (5% for induction, 2–3% for maintenance) and analgesic treatment with Metamizol (100 mg/kg body weight, i.v., Vetalgin, MSD). Rats were ventilated and relaxed (1.5-mg/kg/h Pancuronium bromide i.v, Inresa). The calcium sensor OGB-1 (Oregon Green 488 BAPTA-1 AM, Invitrogen by Thermofisher) was intracranially injected into primary motor cortex (M1) for monitoring of brain state as described in detail by Schmid et al. (2016). Subsequently, a 200-μm optic fiber (Thorlabs) was implanted above the injection site and glued to the skull.

For rs-fMRI and simultaneous Ca^2+^-recordings, anesthesia was switched from Isoflurane to Neurolept by i.p. application of Fentanyl-Droperidol (15-μg/kg Fentanyl, Rotexmedica and 1.5-mg/kg Droperidol, Xomolix, Kyowa Kirin, every 20 min). During the experiment, breathing rate, expiratory CO_2_, and rectal temperature were monitored and kept in physiological ranges. Rs-fMRI data were acquired at 9.4 T (Bruker Biospin MRI GmbH) using a gradientecho echo planar imaging sequence (GE-EPI) with TR 1 s, TE 18 ms, resolution 0.32 × 0.35 mm^2^, slice thickness 1.2 mm, 9 contiguous slices, and 30-min scan time per data set.

**Figure 2:**
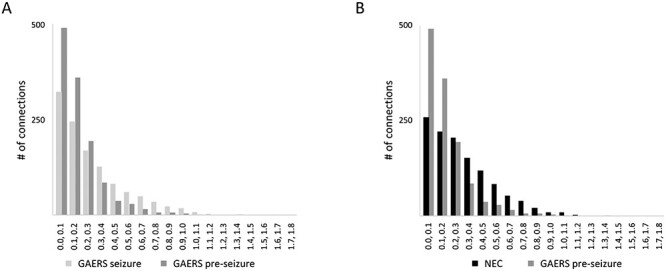
Histograms of *z*-values of the correlation coefficients between individual nodes (brain regions). (*A*) When comparing GAERS before (dark grey) and during seizures (light gray), a significant right-shift toward higher *z*-values during seizures indicated higher functional connectivity in the seizure state. (*B*) When comparing GAERS preseizure (dark gray) versus NEC (black), a significant right-shift toward higher *z*-values in NECs indicated higher functional connectivity in NEC. Mann–Whitney U-test, *P* < 0.001. The total number of connections was in the same range in all groups (GAERS preseizure 1234, GAERS seizure 1148, NEC 1186). GAERS preseizure, number of animals *n* = 9, number of data sets *N* = 12; GAERS seizure, *n* = 8, *N* = 14; NEC, *n* = 7, *N* = 17.

While isoflurane suppresses seizures, previous studies demonstrated that SWDs under Neurolept anesthesia (Pinault et al. 1998) are similar to SWDs occurring in nonanesthetized animals (Danober et al. 1998) in terms of spatial and temporal distribution. After switching to Neurolept, SWDs can usually (based on previous unpublished experiments) be detected within ~90 min after discontinuation of Isoflurane. In GAERS (*n* = 13), one or two rs-fMRI measurements were performed under Neurolept anesthesia before seizures’ onset (preseizure state). SWD onset was identified in Ca^2+^-recordings (Fig. 1*B*), and 1 or 2 rs-fMRI measurements were performed subsequently during seizures (seizure state). NEC (*n* = 7) underwent the same procedure and 30-min rs-fMRI data were acquired ~90 min after switching to Neurolept. Complete data sets (rs-fMRI and Ca^2+^-recordings) from three different groups were used for analysis: GAERS preseizure: number of animals *n* = 9, number of data sets *N* = 12; GAERS seizure: *n* = 8, *N* = 14; NEC: *n* = 7, *N* = 17 (details in Supplementary Table 1).

### rs-fMRI Data Analysis

#### Preprocessing

Raw data were realigned and resliced with SPM12 (http://www.fil.ion.ucl.ac.uk/spm, based on version MATLAB 2018b, Mathworks). The first 15 min of raw data were used. In case any shift in rat head position was detected, continuous 15-min epochs with stable translation (<0.2 mm) and rotation (<0.3°) parameters were selected from 30-min data sets. For data quality assurance, a cortical mask in slice 6 was used to estimate temporal SNR (Supplementary Table 3). Subsequent processing was done with MagnAn (Biocom), an MRI image analysis software based on IDL (© 2020 Harris Geospatial Solutions, Inc). 2D Gaussian smoothing with a kernel size of 3 × 3 pixel, full width half max 0.6 mm was applied and time series were low-pass filtered using a Fourier filter of 0.1 Hz. Brains were manually masked and the global signal mean was regressed out to highlight the changes through the time course. A template of 72 brain regions (Supplementary Table 4; Kreitz et al. 2018) based on the Paxinos and Watson rat brain atlas (Paxinos and Watson 2007) was registered to each individual data set.

### Network Analysis

If not stated otherwise, subsequent analysis was performed using MagnAn. Pearson correlation coefficients of each rs-fMRI-data set were calculated between pairs of brain regions. This procedure led to a symmetric 72 × 72 cross correlation matrix, representing undirected, weighted functional connections between brain regions. Weights represent respective magnitudes of correlational interactions. Only positive correlation coefficients were considered. If several rs-fMRI-data sets were acquired during one brain state, correlation matrices were averaged per animal. Pearson’s *r*-values were converted to Fisher’s *z*-values to provide normal distribution, averaged, and converted back to Pearson’s *r*-values. Group connectivity matrices are displayed in Supplementary Figure 1. The fact that the diagonals representing the interhemispheric connections between regions are clearly visible indicates that the analysis established meaningful functional connections despite the anatomical distance. We extracted connectivity strengths between the cingulate area (cg) and retrosplenial cortex (RS), representing a central feature of the rodent Default Mode Network. Robust connectivity above the significance level (*P* < 0.05, fdr corrected) in the NEC control group (Supplementary Table 3) confirmed sensitivity to functional connectivity. Low connectivity strength between cg and primary sensory cortex (S1HL and S1r) on the other hand verified specificity to functional connectivity (Supplementary Table 3; Grandjean et al. 2020). *Z*-values of correlation coefficients were compared at group level.

### Global Parameters

We calculated global network parameters, clustering coefficient, shortest path length, and small world index (Watts and Strogatz 1998; Humphries and Gurney 2008). Network parameters depend on the network density *k*, with *k* representing the ratio of the actual number of edges to the total number of possible edges between all nodes in the network. Metrics were normalized by comparison to 1000 random networks with the same number of nodes and edges.

The clustering coefficient measures the proportion of connected neighbors of a node with regard to all its possible neighbors. The global clustering coefficient γ is the average of all local clustering coefficients compared with the random network. The global shortest path length λ indicates the efficiency of the overall information flux within the network and is defined by the number of edges needed to travel from one node to another, averaged over all nodes compared with the random network. The small world index σ is the ratio of the latter two metrics, γ/λ, indicating how efficient a network is. Small worldness is >1 for networks with tightly interconnected clusters of nodes, like regular networks, but also with short path lengths between elements, like random networks (Humphries and Gurney 2008). We assessed community structure with Gephi, an open-source software for graph theoretical analysis (Bastian and Heymann 2009) The algorithm implemented herein (Blondel et al. 2008) subdivides the network into subcommunities of densely connected nodes, with nodes belonging to different communities being only sparsely connected.

### Local Parameters

Local parameters were calculated with Magnan at a network density threshold of on average 10 connections per brain region, which led to 360 (strongest) edges in total (*k* = 10, referred to as top 360 connections). At this density, all networks were fully connected. We determined clustering coefficient, shortest path length, degree, strength, and hub score for each node and data set. The degree specifies the number of connections associated with one node. The strength is the sum of link weights of a node. A high hub score index indicates the importance of brain regions with above average high number of connections (degree), a high level of betweeness centrality, and short average path length (Kleinberg 2000).

### Visualization of Network Topology

For visualizing network topology, the software Gephi was used. Nodes were arranged according to the force-based algorithm implemented in Gephi (Jacomy et al. 2014) so that strongly connected nodes appear close together. Node color indicates community affiliation. Node labels identify anatomical brain regions. Group-averaged strengths were additionally calculated in Gephi and used for coding node size in network depictions. The thickness of the edges scales with strength of correlation coefficients.

### Immunohistochemical Staining

Female rats (age 90–150 days, *n* = 3, GAERS and NEC, respectively) were transcardially perfused with 4% (w/v) phosphate-buffered paraformaldehyde (PFA). After removing from the skull, brains were fixed overnight in 4% PFA and later in 30% (w/v) sucrose for 48–72 h. Immunohistochemical staining was performed on free-floating 40-μm-thick coronal sections. First, sections were rinsed three times (10 min) in phosphate-buffered saline (PBS). Then, slices were incubated for 2 h in PBS supplemented with normal goat serum (10% [v/v]), Triton-X100 (0.3% [w/v]), and bovine serum albumin (3% [w/v]). Finally, sections were incubated overnight at 4 °C in primary antibodies: monoclonal mouse anti-parvalbumin (PV; 1:500) and monoclonal mouse anti-neuronal nuclear protein (NeuN; 1:1000). After incubation with one of the primary antibodies, sections were washed three times for 10 min in PBS and then transferred to the secondary antibody solution (Alexa Fluor 488 goat anti-mouse-IgG, 1:1000) for 2 h. In the last step, sections were washed three times for 10 min in PBS and mounted with a mounting medium containing the nuclear marker 4′,6-diamidino-2-phenylindole (VECTASHIELD Mounting Medium with DAPI, Vector Laboratories) for confocal microscopy (Nikon eC1plus) equipped with ×10 objective (Leica). DAPI staining was used to define boundaries and layers of the granular RS; 40-μm-thick slices were scanned with 2-μm *Z*-stack steps and then combined together as one picture. Cells were counted in a 960 × 760 μm field of view in 3 slices from each animal with ImageJ (Wayne Rasband, National Institutes of Health).

### Experimental Setup for Ex Vivo Slice Experiments

#### Preparation of the Granular Retrosplenial Cortex Slices

Experiments were performed on NEC (*n* = 13) and GAERS (*n* = 12) ranging in age from postnatal day P105–P120. After decapitation, the skull cap was surgically removed caudal to Bregma and a block of brain tissue containing the RS was submerged in ice-cold aerated (O_2_) saline containing (in mM): sucrose, 200; PIPES, 20; KCl, 2.5; NaH_2_PO_4_, 1.25; MgSO_4_, 10; CaCl_2_, 0.5; dextrose, 25; pH 7.35 was set with NaOH. Coronal slices (250–300 μm thick) were prepared with a vibratome. Slices were transferred to a holding chamber and kept submerged (at 30 °C for 30 min, thereafter at room temperature) in artificial cerebrospinal fluid (ACSF) containing (in mM): NaCl, 125; KCl, 2.5; NaH_2_PO_4_, 1.25; NaHCO_3_, 24; MgSO_4_, 2; CaCl_2_, 2; dextrose, 10; pH was adjusted to 7.35 by aerating the solution with carbogen (95% O_2_ and 5% CO_2_ gas mixture).

### Patch Clamp Method

The granular RS was visualized by using differential interference contrast infrared video microscopy with the camera (Kappa) connected to an upright microscope (ZEISS Axio Examiner.D1). All experiments were done in deep layer (Layer 5) of the cortex at 30–32 °C. Pyramidal neurons of Layer 5 were visually identified (somatic size was usually more than 20 μm).

Membrane currents were measured with glass microelectrodes pulled from borosilicate glass capillaries (GC150T-10; Clark Electromedical Instruments) with a resistance of 2.5–3.5 MΩ. Patch electrodes were connected to a HEKA EPC-9 patch clamp amplifier (HEKA Elektronik) via a chlorinated silver wire. Access resistances were between 8 and 20 MΩ. Series resistance compensation of ≥50% was routinely applied. Experiments were controlled by the PatchMaster recording software (HEKA Elektronik) at a sampling rate of 10 kHz, with a low-pass filter of 2–3 kHz. All recordings were corrected offline for a liquid junction potential of 10 mV (*V*_M_ = *V*_P_—10 mV; with *V*_M_ = membrane potential and *V*_P_ = pipette voltage).

### Voltage Clamp Recordings

Spontaneous synaptic events were measured in voltage clamp mode. During recordings slices were continuously oxygenated and perfused (~2 mL/min) with ACSF containing (in mM): NaCl, 125; KCl, 2.5; NaH_2_PO_4_, 1.25; NaHCO_3_, 24; MgSO_4_, 2; CaCl_2_, 2; dextrose, 10; pH was adjusted to 7.35 by bubbling the solution with carbogen. Spontaneous excitatory postsynaptic currents (sEPSCs) were recorded from tissue slices of 3 GAERS and 4 NEC in the presence of GABA receptor blockers (10-μM CGP and 25-μM gabazine) at −60 mV. The micropipette was filled with a potassium-gluconate (K-gluconate)-based intracellular solution containing (in mM): K-gluconate, 88; K_3_-citrate, 20; NaCl, 10; HEPES, 10; MgCl_2_, 1; CaCl_2_, 0.5; BAPTA, 3; Mg-ATP, 3; Na_2_-GTP, 0.5. The pH of 7.25 was set with KOH. Osmolality of the internal solution was 295 mOsm/kg. Spontaneous inhibitory postsynaptic currents (sIPSCs) were measured from 3 GAERS and 3 NEC and were pharmacologically isolated using glutamatergic receptor blockers (20 μM AP-5 and 10 μM DNQX) at −60 mV. The internal solution contained a high molarity of Cl^−^ (in mM): KCl, 110; NaCl, 10; EGTA, 11; HEPES, 10; Phosphocreatin, 15; Mg-ATP, 3; Na-GTP, 0.5; MgCl_2_, 1; CaCl_2_, 0.5. pH was adjusted to 7.25 with KOH. Osmolality was set at ~290 mOsm/kg. Spontaneous currents were recorded for 5 min (30-s sweep duration, repeated 10 times). The frequency and the amplitude of the events were analyzed offline manually using Clampfit (v. 10.7; Molecular Devices) software.

### Current Clamp Recordings

Current clamp method was used to study the electrical properties of the pyramidal neurons. Recordings were done in brain slices of 6 GAERS and 6 NEC in ACSF (as described above). Micropipettes were filled with K-gluconate-based intracellular solution (as described above). The resting membrane potential (RMP) was measured in current clamp mode immediately after opening the membrane with zero current injection. Recordings were done at RMP and at −60 mV. To measure active and passive properties of the electrical membrane, 1000-ms hyperpolarizing (up to −240 pA) and depolarizing (up to 360 pA) current steps with 40-pA increments were injected as square-wave current pulses. Analysis was done offline using FitMaster software (HEKA Elektronik). Membrane time constants (τ_m_) were obtained by fitting negative voltage deflections (induced by hyperpolarizing current injections of −40 pA) to single or double exponentials. Input resistance (*R*_in_) was calculated according to Ohm’s law: *R*_in_ = Δ*V/I*. The membrane capacitance was estimated using the following formula: *C*_m_ = τ_m_/*R*_in_. The anomalous rectification (or voltage sag amplitude) of the current injection at −240 pA was calculated as the differences between the maximal (at the beginning of the hyperpolarizing current injection) and steady state voltage deflection (at the end of the hyperpolarizing current injection). For depolarizing current steps, the number of action potentials (APs) was determined. For the spike in a train of APs, threshold and amplitude were determined. In addition, the number of rebound spikes, that is, the number APs following release from hyperpolarizing, was determined.

**Figure 3:**
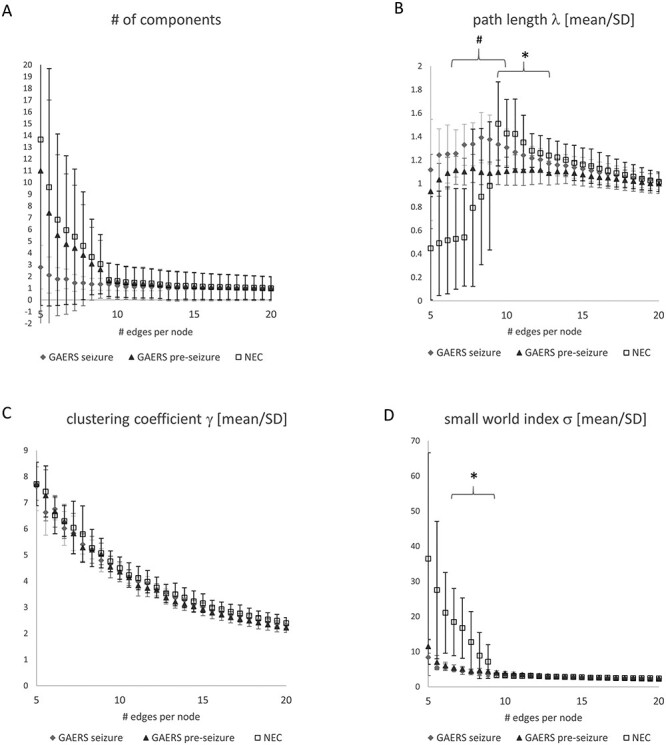
Average global network parameters of GAERS in preseizure and seizure state and of NEC plotted versus the number of edges per node. (*A*) At a network density of 10 edges per node, networks were fully connected (number of components ~1). At this network density, average path length (*B*) was highest for NEC, followed by GAERS during seizures, and lowest for GAERS preseizure (*P < 0.05 vs. NEC, #*P* < 0.05 vs. GAERS seizure). Average clustering coefficient (*C*) and average small world index (*D*) did not differ between groups at a network density of 10 edges per node. GAERS preseizure, *n* = 9, *N* = 12; GAERS seizure, *n* = 8, *N* = 14; NEC, *n* = 7, *N* = 17.

### Statistics

#### Rs-fMRI

Global parameters were compared on group level with unpaired *t*-test. To highlight group differences in functional connectivity in GAERS between preseizure and seizure state and between GAERS preseizure state and NEC, a homoscedastic *t*-test between false discovery rate (fdr)-corrected correlation matrices was performed. Network based statistics (NBS) were applied by correcting this *t*-test for multiple testing by 1000 times permutation (Zalesky et al. 2010). For comparison of local node parameters, we used a two-factorial ANOVA with interaction (IBM SPSS statistics 25). Obvious group differences in local node parameters average path length and strength were then tested for statistical significance by applying post hoc *t*-test. Due to the fact that consecutive preseizure and seizure recordings were only available for three animals (see details in Supplementary Table 1), we used unpaired *t*-test.

#### Immunohistochemistry and Slice Experiments

Statistical analysis was performed with IBM-SPSS (version 24). All results are presented as mean ± SEM. For patch clamp recordings, numbers given in text (*N*) refer to numbers of neurons and for immunostaining—(*n*) to numbers of animals. Statistical differences between groups were established with repeated measures ANOVA, independent samples, two-tailed *t*-test with post hoc Tukey’s multiple comparisons test. Significance levels are indicated as *P* values. *, **, and *** indicate *P* < 0.05, *P* < 0.01, and *P* < 0.001, respectively.

## Results

In this study, we explored brain networks in GAERS rats, a rat model of spontaneous absence epilepsy. We analyzed rs-fMRI data acquired, first, before start of SWDs (preseizure state) and second, after SWD onset (seizure state) (Fig. 1*A*). Applying Neurolept anesthesia, all but one rat reliably experienced seizures during examinations (Supplementary Table 1). Simultaneous, optical Ca^2+^-recordings allowed detection of brain state change 78–188 min (123 ± 35 min, mean ± SD) after switching from Isoflurane to Neurolept. In seizure state, SWD epochs alternated with short epochs of rest (Fig. 1*B* upper row) and persisted during acquisition of seizure state data. Data from nonepileptic controls (NEC) were acquired under the same anesthetic regimen. Power spectra of the Ca^2+^-traces confirmed the exclusive occurrence of typical SWDs at ~6 Hz in GAERS during seizures (Fig. 1*B* lower row).

### Global Network Topology

To investigate brain networks, functional connectivity was calculated from rs-fMRI data and analyzed by graph theoretical methods. Average group correlation matrices are displayed in Supplementary Figure 1. Interhemispheric connections between regions were clearly represented by diagonals in the matrices, indicating that meaningful functional correlations were obtained. Connectivity was lower in the preseizure state when compared with the seizure state (Fig. 2*A*). Likewise, connectivity was lower in the preseizure state when compared with NEC (Fig. 2*B*), as indicated by the right-shift of *z*-values of the correlation coefficients between individual nodes (=brain regions, Mann–Whitney U-test, *P* < 0.001). Global network parameters (Fig. 3) depend on network density *k* that was used for the analysis procedure. At *k* = 10, all networks were fully connected (number of components ~1, Fig. 3*A*). We therefore used a network density of 10 edges per node to compare global and local node parameters of fully connected networks. In GAERS, average shortest path length (Fig. 3*B*) was higher during seizures compared with preseizure state (unpaired *t*-test, preseizure vs. seizure state *P* < 0.05). Compared with NEC, shortest path length was low (unpaired *t*-test GAERS preseizure vs. NEC, *P* < 0.05). Global clustering coefficient (Fig. 3*C*) and small world index (Fig. 3*D*) were similar in all groups at *k* = 10.

### Communities

**Figure 4:**
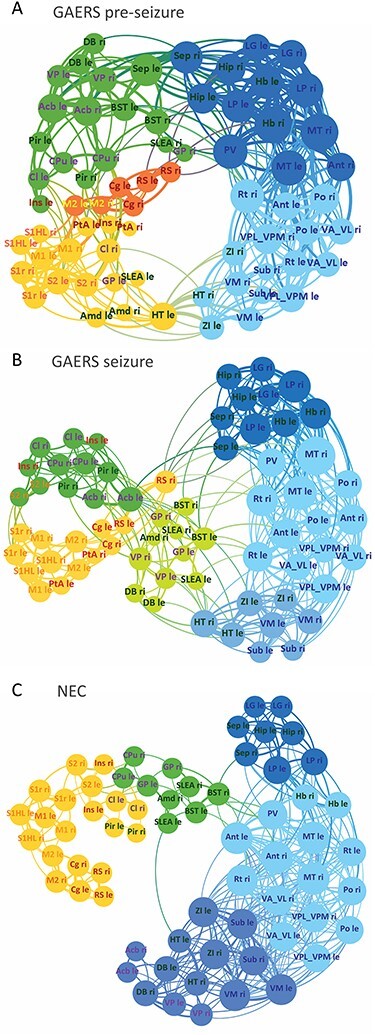
Force-based plots of average resting state networks of GAERS in preseizure (*A*) and seizure (*B*) state and of NEC (*C*) at a network density of *k* = 10. (*A*) The preseizure network subdivided into mostly cortical (yellow and orange), basal ganglia/limbic (dark and light green), and thalamic/limbic (two shades of blue) communities. (*B*) During seizures, cortical brain regions united in one (yellow) community. Basal ganglia/limbic regions segregated into two, a primarily basal ganglia (dark green) and a primarily limbic (light green) community. Thalamic/limbic regions separated in three communities (different shades of blue). (*C*) In NEC, all cortical regions assembled in one community (yellow). Basal ganglia and limbic regions formed one (green) community. Thalamic/limbic regions arranged in three communities (different shades of blue). Node colors represent community affiliation. Color of region labels indicates functional group: orange—sensorimotor, red—association cortex, green—limbic system, blue—thalamus, and lilac—basal ganglia. Node size scales with local strength. Abbreviations for brain regions are listed in Supplementary Table 3. GAERS preseizure, *n* = 9, *N* = 12; GAERS seizure, *n* = 8, *N* = 14; NEC, *n* = 7, *N* = 17.

Network topology was analyzed by generating force-based plots of nodes, arranging brain regions into distinct but interacting communities. Overall, largely similar communities were observed, according to functional group affiliation of the contained nodes (Fig. 4). Mainly cortical regions, sensorimotor and association cortex, gathered in one (yellow, GAERS seizure and NEC) or two (yellow and orange, GAERS preseizure) communities. Basal ganglia and limbic regions formed one (GAERS pre-seizure and NEC) or two (GAERS seizure) communities, depicted in green. Thalamic regions alone or gathering with limbic regions were found in two (GAERS pre-seizure) or three (GAERS seizure and NEC) closely connected communities, depicted in different shades of blue, respectively. Brain regions of both hemispheres were often located in the same community, indicating a strong left–right connectivity.

A closer look at community structure of GAERS in preseizure and in seizure state (Fig. 4*A* vs. *B*) indicated short-term changes of the brain network associated with the acute change of brain state. Most cortical regions arranged in one community during seizures. The basal ganglia/limbic community (green), formerly found in the preseizure state, segregated into two communities (light and dark green) in the seizure state. Thalamic/limbic regions (blue) segregated from two into three, one purely thalamic and two thalamic/limbic communities, still showing many connections during seizures. The number of communities increased from 5 to 6, in line with the higher global shortest path length during seizures compared with the preseizure state.

Community structure also differed between strains (Fig. 4*A* vs. *C*). In NEC, all cortical regions strictly gathered in one community. In GAERS preseizure, retrosplenial cortex (RS), cingulate cortex, and secondary motor cortex formed a separate community (orange), and other cortical regions assembled with basal ganglia and limbic regions (yellow). Thalamic/limbic regions (blue) arranged in three communities in NEC and in two communities in GAERS.

### Connections between Brain Regions

**Figure 5:**
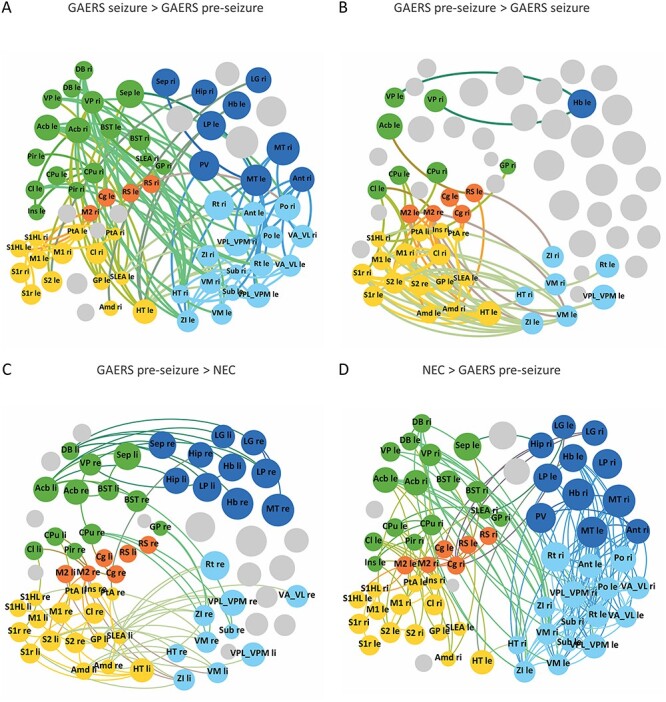
Display of significantly altered connections between groups, separated by signature of difference, and mapped onto the preseizure community structure. NBS was based on fdr-thresholded data at α = 0.05. (*A*) When comparing between brain states (upper row, component size: 64 nodes, 175 connections, component probability: *P* < 0.001), stronger connections were found within cortical regions (yellowish), within thalamic (blueish), and between thalamic and limbic regions (greenish) in seizure state. (*B*) In preseizure state, stronger connections were mainly observed between cortical and thalamic/limbic regions (light green) and between basal ganglia regions and limbic/cortical regions. (*C*) When comparing between rat strains (lower row, component size: 68 nodes, 245 connections, component probability: *P* = 0.004), stronger connections were found between basal ganglia and limbic regions and between thalamic and limbic regions in GAERS preseizure. (*D*) NEC had stronger connections within thalamic (blueish) and between thalamic and limbic regions and within association cortex and limbic/thalamic regions (greenish). Node colors represent communities. Only nodes with statistically different connections are shown in color. Gray nodes did not have significantly different connections. Abbreviations for brain regions are listed in Supplementary Table 3. GAERS preseizure, *n* = 9, *N* = 12; GAERS seizure, *n* = 8, *N* = 14; NEC, *n* = 7, *N* = 17.

During seizures, the cortical communities (yellow and orange) segregated from the remaining brain network (Fig. 5*A*) and showed many significantly stronger (α = 0.05, fdr corrected, *P* < 0.001) intracortical connections compared with the preseizure state. NBS furthermore indicated that during seizures, particularly connections within and between basal ganglia and limbic regions (green) and within thalamic regions (blue) were significantly stronger. In the preseizure state (Fig. 5*B*), connections between cortical regions and basal ganglia and limbic regions were stronger.

When comparing between strains (Fig. 5, lower row), GAERS exhibited significantly stronger (α = 0.05, fdr corrected, *P* < 0.004) intracortical connections (Fig. 5*C*) compared with NEC. Furthermore, basal ganglia exhibited stronger connections with cortical, limbic, and thalamic regions. NEC primarily formed significantly stronger connections within thalamic regions and between limbic regions and association cortex.

### Local Node Parameters

To further assess the role of individual brain regions, region-specific local network parameters were statistically analyzed (Table 1). In agreement with average global parameters, no significant differences in average local clustering coefficients were detected between brain states or rat strains.

**Table 1:** ANOVA analysis of local network parameters

Factor		df	Clustering coefficient	Shortest path length	Degree	Strength	Hubscore
Group	*F*	2	4.233	50.000	0.040	14.083	0.010
	*P*		0.015	**<0.001**	0.961	**<0.001**	0.999
Brain region	*F*	71	1.981	3.464	12.765	10.968	14.525
	*P*		**<0.001**	**<0.001**	**<0.001**	**<0.001**	**<0.001**
Interaction	*F*	140	1.049	1.017	1.660	1.990	1.096
	*P*		0.338	0.434	**<0.001**	**<0.001**	0.219

Notes: Results of factorial ANOVA with interactions for comparison of local node parameters. The factor brain region was significant for all parameters, indicating node-specific differences in networks. The factor group was significant for shortest path length and strength. Significant interaction for degree and strength indicated group-dependent shift of node parameters. Only significance level *P* < 0.01 is highlighted in bold, because Levene test indicated non-equality of variances. Post hoc *t*-tests were performed for selected brain regions (Fig. 6 and Supplementary Table 5). df = degree of freedom. GAERS pre-seizure, *n* = 9, *N* = 12; GAERS seizure, *n* = 8, *N* = 14, NEC, *n* = 7, *N* = 17.

**Figure 6:**
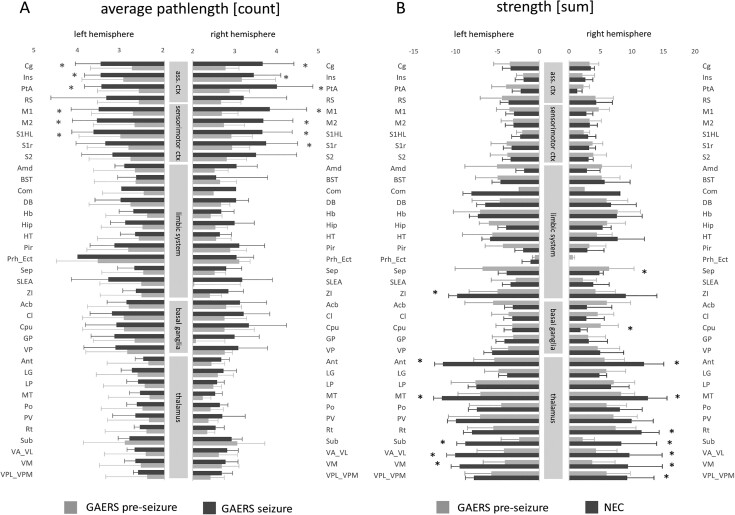
Average local node parameters path length and strength sorted by functional group and hemisphere. (*A*) Association cortex and sensorimotor cortex regions showed significantly higher average path lengths during seizures (dark gray) when compared with the preseizure state (light gray). Shortest path lengths of all other brain regions were similar in both brain states. (*B*) Septum (Sep) and striatum (Cpu) exhibited significantly higher strength in GAERS (light gray) compared with NEC (dark gray). Several thalamic regions and limbic region. Zona incerta (Zi) showed higher local strength in NEC compared with GAERS. Bars show mean ± SD. Asterisk indicate significant difference, post-hoc *t*-test, *P* < 0.05, uncorrected. Abbreviations for brain regions are listed in Supplementary Table 4. GAERS preseizure, *n* = 9, *N* = 12; GAERS seizure, *n* = 8, *N* = 14; NEC, *n* = 7, *N* = 17.

In line with the observed cortical segregation during seizures, average shortest path lengths (Fig. 6*A*) of association cortex and sensorimotor cortex regions were longer in seizure state compared with preseizure state. Average shortest path lengths of all other brain regions were similar between brain states. Neither degrees, strengths, nor hub scores significantly differed between brain states. When comparing between rat strains, NEC had higher strengths (Fig. 6*B*) in a number of thalamic nuclei and in the limbic region Zona incerta (Zi). In contrast, strength was higher in septum and striatum in GAERS.

**Figure 7:**
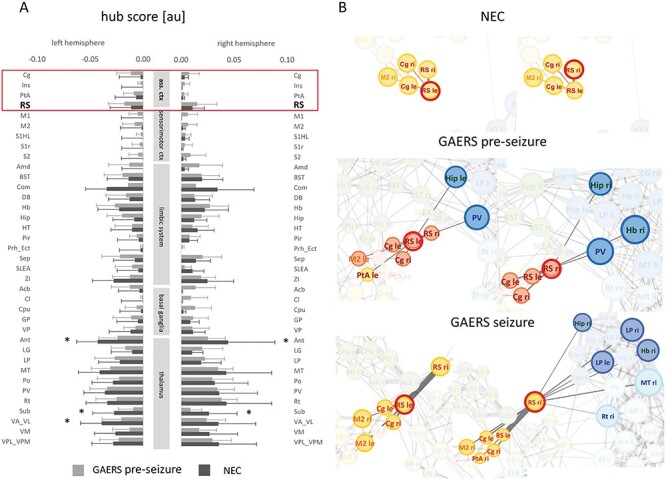
(*A*) Average hub scores per brain region. Hub scores were lowest in cortical regions and highest in thalamic regions. In GAERS, RS hub scores were above cortical group average. Regions of the association cortex are framed in the red box. Thalamic regions, Ant, Sub, and VA_VL exhibited lower hub scores compared with NEC (unpaired *t*-test, *P* < 0.05). (*B*) Zoomed force-based network plots centered onto retrosplenial cortex. Under the top 360 edges, only connections within cortical community were apparent in NEC. In GAERS, independent of brain state, connections reach out to hippocampus and thalamic regions. During seizure state, data suggested a tendency to lateralization. Node colors represent community affiliations similar to Figure 4. Color of region labels indicates functional group: orange—sensorimotor, red—association cortex, green—limbic system, and blue—thalamus. Node size scales with local strength. Abbreviations for brain regions are listed in Supplementary Table 3. GAERS preseizure, *n* = 9, *N* = 12; GAERS seizure, *n* = 8, *N* = 14; NEC, *n* = 7, *N* = 17.

Within different functional groups specific nodes showed above community-average hub scores (Fig. 7*A*): In GAERS, RS and limbic regions including habenuli (Hb), hippocampus (Hip), and septal area (Sep) stood out. Zona incerta and the thalamic regions anterior thalamic nucleus (Ant), submedial nucleus, and nucleus ventralis anterolateralis (VA_VL) had hub status in NEC (unpaired *t*-test, *P* > 0.05). RS showed distinct connectivity patterns (Fig. 7*B*) when comparing seizure state in GAERS, preseizure state in GAERS and NEC. While in NEC, RS were only connected intracortically, in GAERS, preseizure correlations to subcortical regions were found in addition. In both cases, patterns were largely similar in both hemispheres of the brain. During seizure state, however, data suggest a tendency to lateralization. While for left RS, only correlations with cortical regions were observed, right RS seemed to extend its links to subcortical regions, as compared with the preseizure state.

### Immunohistochemistry of Retrosplenial Cortex

**Figure 8:**
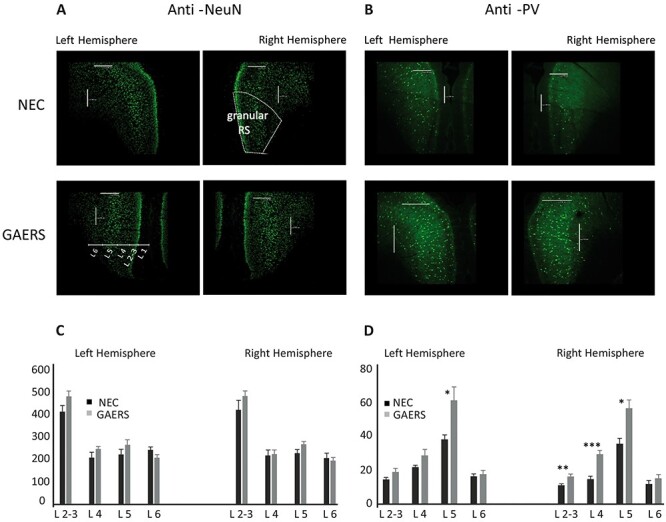
Immunohistochemistry of the retrosplenial cortex. (*A*) Anti-NeuN antibody staining in NEC (upper panels) and GAERS (lower panels). (*B*) Anti-PV antibody staining in NEC (upper panels) and GAERS (lower panels). Scale bars 240 μm. (*C*) Number of NeuN-positive neurons presented in percentage in the left and right hemispheres. (*D*) Number of PV-positive neurons presented in percentage in the left and right hemispheres. *P < 0.05. NEC, *n* = 3; GAERS *n* = 3.

To investigate the cellular basis of a potentially altered role of RS, we compared the number of neurons in RS of NEC and GAERS by using immunohistochemical staining with anti-NeuN antibody (Fig. 8). This approach was motivated by the previous finding that during limbic seizures, there was a loss of neurons in layers 4 and 5 in granular RS of Wistar rats (Cardoso et al. 2008). In general, we found that layer (L) 1 was mostly devoid of neurons, while a population of small, nonpyramidal shaped and densely packed neurons characterized L 2 and 3. L 4 and 6 had small pyramidal neurons. L 5 contained large sized pyramidal neurons and was readily distinguishable from the other layers (Fig. 8*A*). Cell numbers were similar in NEC and GAERS, both in left and right hemisphere (Fig. 8*C*).

Initiation and propagation of epileptic discharges in the cortex are partially determined by the local synaptic connectivity of coupled populations of glutamatergic and GABAergic neurons (Chizhov et al. 2019). In RS, pyramidal neurons receive inhibitory synapses mostly from fast spiking PV+ interneurons of the same layer, thereby regulating their excitability (Sempere-Ferràndez et al. 2019). Therefore, we next analyzed the pattern of PV immunoreactivity in RS (Fig. 8*B*). In all layers except L 1, PV+ cells were detected, with highest numbers in L 4 and 5. Cell numbers were similar in left hemisphere of NEC and GAERS, except for L 5, with significantly more PV+ interneurons in GAERS. In all but L 6 of the right hemisphere, significantly more PV+ interneurons were found in GAERS (Fig. 8*D*).

### Properties of Excitatory and Inhibitory Inputs to Layer V Pyramidal Cells in Granular Retrosplenial Cortex

**Figure 9:**
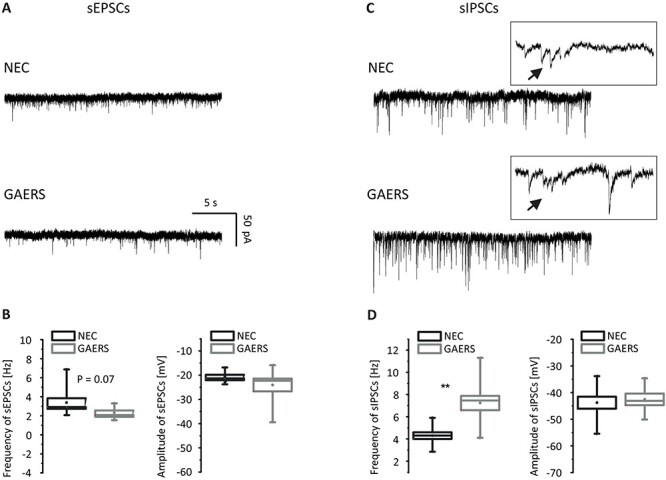
Voltage clamp recordings of pyramidal neurons of granular retrosplenial cortex. (*A*) Example traces of sEPSCs from NEC (upper panel) and GAERS (lower panel). (*B*) Frequencies (left panel) and amplitudes (right panel) of sEPSCs of NEC (dark gray, *N* = 4) and GAERS (light gray, *N* = 3). (*C*) Example traces of sIPSCs from NEC (upper panel) and GAERS (lower panel). (*D*) Frequencies (left panel) and amplitudes (right panel) of sEPSCs of NEC (dark gray, *N* = 3) and GAERS (light gray, *N* = 3). **P < 0.01.

In order to assess whether graph theoretical network differences were associated with changed synaptic activity in RS neurons, L 5 pyramidal cells were recorded under voltage clamp conditions. The RS has dense reciprocal connections with Ant and hip from where it receives excitatory and inhibitory projections, respectively. Therefore, we investigated spontaneous synaptic events. sEPSC measurements were done in ACSF in the presence of GABA receptor blockers (CGP, gabazine) (Fig. 9*A*). While there were no apparent differences in average amplitude between strains (NEC: −20.6 ± 0.8 pA, *n* = 9; GAERS: −24.1 ± 2.7 pA, *n* = 8; F = 4.9, df = 8, *P* = 0.2), we observed a tendency toward higher sEPSC frequencies in NEC (3.4 ± 0.5 Hz, *n* = 9) compared with GAERS (2.3 ± 0.2 Hz, *n* = 8; *F* = 0.6, df = 12, *P* = 0.07) (Fig. 9*B*). sIPSCs were studied in the presence of glutamate receptor blockers (APV, DNQX) (Fig. 9*C*). The amplitude of inhibitory currents did not differ significantly between the groups, but frequencies of sIPSCs were significantly higher in GAERS (7.2 ± 0.6 Hz, *n* = 9; *F* = 1,4, df = 11, *P* = 0.001) in comparison to NEC (4.3 ± 0.3 Hz, *n* = 10; Fig. 9*D*). In addition, most events of sIPSCs from both groups appeared to be polysynaptic (inserted traces in Fig. 9*C*).

### Current Clamp Recordings

**Figure 10:**
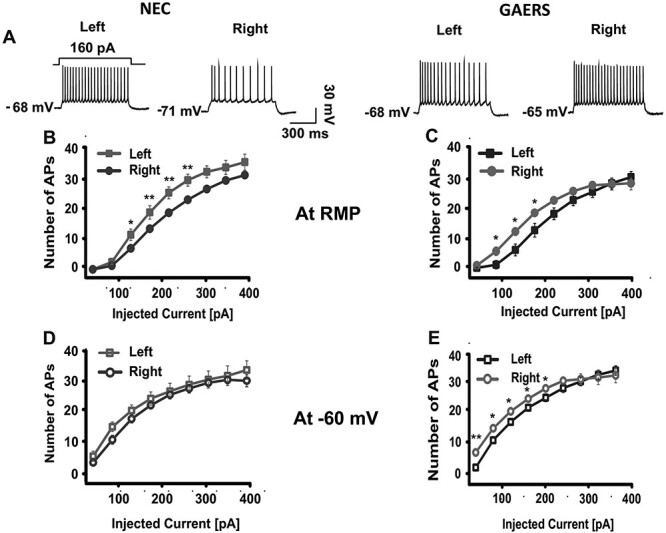
Current clamp recordings of pyramidal neurons of granular RS. (*A*) Firing pattern of pyramidal neurons in granular retrosplenial cortex from NEC (left) and GAERS (right) at +160 pA current injection. (*B*–*E*) Input–output curves show the number of APs at RMP (*B*, NEC, *N* = 3, GAERS, *N* = 3; *C*, NEC, *N* = 5, GAERS, *N* = 5) and at −60 mV (*D*, NEC, *N* = 3, GAERS, *N* = 3; *E*, NEC, *N* = 5, GAERS, *N* = 5) in left and right hemisphere, respectively. *P < 0.05, **P < 0.01.

To determine the intrinsic membrane properties of L 5 pyramidal neurons in NEC and GAERS, we performed current clamp measurements in the RS of left and right hemisphere at RMP and −60 mV (Fig. 10*A*). Neurons from both strains showed similar RMPs in left (NEC: −68.8 ± 1.1 mV, *n* = 10; GAERS: −66.6 ± 0.9 mV, *n* = 11; *F* = 0.02, df = 18, *P* = 0.2) and right (NEC: −68 ± 0.6 mV, *n* = 22; GAERS: −67.1 ± 0.8 mV, *n* = 24; *F* = 2.4; df = 42, *P* = 0.4) hemispheres. When challenged with positive current steps, pyramidal neurons from NEC revealed higher firing rates in the left compared with right hemisphere (Fig. 10*B*). In contrast, pyramidal neurons from GAERS revealed higher firing rates in the right compared with the left hemisphere. Similar differences were noticed when the membrane potential was set at −60 mV (only significant for GAERS). This can be explained by changes in input resistance (*R*_in_). *R*_in_ was significantly higher in the right hemisphere of epileptic rats both at RMP (GAERS: 140.6 ± 6.6 MΩ, *n* = 24; NEC: 113.6 ± 5.7 MΩ, *n* = 22; F = 0.5, df = 41, *P* = 0.004) and at −60 mV (GAERS: 195 ± 9.6 MΩ, *n* = 18; NEC: 161 ± 6.6 MΩ, *n* = 20; *F* = 0.8, df = 31, *P* = 0.005). Except membrane time constant (τm), passive and active membrane properties, such as membrane capacitance (Cm), membrane time constant (τm), voltage sag amplitude, AP threshold and AP amplitude, and number of rebound spikes, were not statistically different between groups under both experimental conditions (Supplementary Table 4).

## Discussion

We applied graph theoretical analysis of rs-fMRI data to characterize brain network changes in the GAERS model of absence epilepsy. We compared data obtained before seizure onset and during seizures in GAERS to identify differences between brain states. We also analyzed data, obtained under the same anesthetic regimen in NEC, to investigate whether frequently occurring absence seizures also chronically modulate the brain network.

On global level, small world topology was preserved independent of brain state and rat strain. Focusing on community structure and further on local node level unraveled potentially epilepsy-relevant network characteristics: The most prominent acute change upon seizure onset was the segregation of cortical regions from the remaining network. We found stronger connections between thalamic regions and with limbic regions during seizures. When comparing between strains, intrathalamic connections were particularly prominent in NEC, indicated by significantly higher local strengths and hub scores. Cortical hub scores were generally much lower. Only retrosplenial cortex had slightly higher, above cortical community-average hub scores. Different connection patterns of the RS in NEC and GAERS suggested a potential role in the epileptic network. Since the RS has not been a focus of previous studies, we choose this brain region for immunohistochemical and electrophysiological investigation in parallel to our network analyses.

The efficiency of network organization in the brain is built upon the strength of correlations between brain regions. We found the lowest global functional connectivity in preseizure state when comparing it either to seizure state or to NECs. In the seizure state, NBS identified many stronger intrathalamic connections, and the local parameter average strength was significantly higher in a number of thalamic nuclei. These findings support the hypothesis of network regularization during seizures, compared to a more random pre-ictal network organization (Ponten et al. 2007; Schindler et al. 2008; Kramer and Cash 2012). When comparing human patients with epilepsy to healthy controls, some clinical fMRI studies reported similar small world indices, although average shortest path lengths and clustering coefficients varied between these studies (Centeno and Carmichael 2014). Others reported a weaker clustering and lower small worldness, indicating a less efficiently organized network (Drenthen et al. 2020) in children with absence epilepsy. In our study, small world indices and global average clustering coefficients did not differ between strains. Only the global average shortest path length was slightly higher in GAERS during seizures compared with the preseizure state. This finding reflected the higher local, cortical shortest path lengths, most likely due to the segregation of cortical regions. An increase of shortest path length in the ictal compared with the pre-ictal state was also found by graph theoretical analysis of surface EEG recordings in children with absence epilepsy (Ponten et al. 2009).

In line with our finding of cortical segregation, multisite Local Field Potential recordings in the WAG/Rij absence epilepsy rat model showed an increase of intracortical interactions during seizures (Sysoeva et al. 2016). High-functional connectivity in cortex was also reported in human childhood absence epilepsy (Masterton et al. 2012). Disrupted interaction among cortical and subcortical systems may be the cause of impairment of consciousness during seizures (Blumenfeld 2012). In our study, more and stronger connections within the cortical community as well as from cortical to basal ganglia and to limbic regions were apparent in GAERS than in NEC. Mishra et al. (2013) have reported similar findings when comparing interictal epochs in WAG/Rij rat to equal-length epochs from age-matched Wistar rats. On the other hand, NEC exhibited strongest interactions within thalamic and between association cortex and limbic/thalamic regions.

In contrast to the general retreat of sensorimotor and association cortex regions, retrosplenial cortex tended to extend connections to noncortical regions in GAERS. In another model of human absence and generalized tonic–clonic epilepsy, the PTZ-induced seizure rat model, RS had an increased signal amplitude, as also observed for Ant and dentate gyrus (Tenney et al. 2003; Brevard et al. 2006). EEG recordings in GAERS (Danober et al. 1998) accordingly indicated that cortex, RS, and thalamic relay nuclei play an important role. Changes in the structure and function of the RS have already been noticed during limbic seizures (Cardoso et al. 2008). fMRI data from patients with generalized idiopathic epilepsy showed significant symmetrical activated clusters in RS during SWD activity (Vaudano et al. 2009). However, the authors did not include RS in their connectivity analysis, believing that it might play a role in spreading and not initiating the pathological activity.

The subtle increase in hub score together with a higher immunoreactivity for PV+ interneurons in GAERS both suggest an altered role of RS in this rat model. The control of cortical network excitability by PV+ interneurons in different forms of epilepsy has been postulated before. In a mutant mouse model devoid of CaV 2.1 channels in cortical interneurons, severe forms of generalized epilepsy including absence, myoclonic, tonic, and tonic–clonic seizures were observed with fast spiking neurons exhibiting impaired GABA release (Rossignol Ethor et al. 2013; Wong 2014). A mouse models of absence epilepsy bearing mutated GABA A receptors revealed reduced cortical inhibition that was localized to the somatosensory cortex (pyramidal neurons in layers 2/3), suggesting a defect in GABAergic interneurons (Tan et al. 2007). Optogenetic manipulations of PV+ and SST+ interneurons in the medial prefrontal cortex have been shown to contribute to spatial working memory (Kim et al. 2007). It is possible that PV+ interneurons in granular RS will also contribute to some extent to spatial learning and memory processes. Stronger immunoreactivity of the PV+ interneurons and inhibitory transmission in granular RS, observed in our experiments, might therefore contribute to declined learning in the reference memory task and impaired working memory in GAERS rats (Marques-Carneiro et al. 2016). It is interesting to note that long-term enhancement of PV+ interneuron-mediated neurotransmission in the RS has been discussed to partially explain the learning and memory deficits found in mice exposed to ethanol during fetal development (Bird et al. 2021). Similar considerations may apply for the learning deficits found in absence epilepsy models and the enhanced inhibitory transmission found in the present study.

In addition, we found increased sIPSCs and decreased sEPSCs frequencies in pyramidal neurons of GAERS. Excitatory inputs to granular RS come from Ant and hip. Inputs from Ant first activate superficial layers and then signal propagates to deep layers (Nixima et al. 2017). In contrast, excitatory axons from hip (via subiculum) directly excite proximal dendrites in deep layers. They form feedforward inhibition, which plays an important role in mnemonic functions in granular RS (Gabriel and Sparenborg 1986). Direct long-range GABAergic inputs to granular RS come from hip and originate from stratum lacunosum-moleculare interneurons (Corcoran et al. 2018). This GABAergic projection is thought to participate in encoding and storage of hippocampal derived information (Vann et al. 2009). Other GABAergic inputs to granular RS derive from local interneurons. Layer 5 pyramidal neurons make excitatory synapses on Layer 5 PV+ interneurons (Lourenço et al. 2014), which in turn cause feedback inhibition of the pyramidal cells. In summary, cortical and subcortical regions, like Ant, appear to mediate the inhibition of cortical regions via RS.

Electrophysiological data presented here point to differences in synaptic activities and cellular excitability in RS of GAERS and NEC. It has been postulated that granular RS together with Ant and hip contribute to spatial learning and episodic memory (Vann et al. 2009) with the deep layers of the granular RS being involved in the integration of sensory and memory information. We noticed that Layer 5 pyramidal neurons in the granular RS, in particular of the right hemisphere, exhibited higher input resistance and revealed higher excitability in GAERS compared with NEC. These results are in line with previous data from somatosensory and motor cortex, where the authors have described enhanced excitability of deep layer cells in GAERS compared with nonepileptic Wistar outbred rats (Polack et al. 2007).

In conclusion, we have shown that graph theoretical network analysis based on rs-fMRI allowed for the identification of acute and chronic brain network changes in the GAERS model. We recognized subtle differences of connectivity pattern of RS, forming more connections beyond cortex in epileptic rats with a tendency to lateralization during seizures. A role as hub between subcortical and cortical regions in epilepsy was supported by increased numbers of PV+ interneurons in granular RS of GAERS together with enhanced synaptic activity, neuronal excitability, and inhibitory transmission in pyramidal neurons. Our result shows that the combination of multimodal fMRI data, graph theoretical methods, and electrophysiological recordings is able to resolve subtle changes in cortex, which none of the tools alone can identify. Altogether, the RS may be a promising target for modulation of seizure activity and/or comorbidities.

## Notes

*Conflict of Interest*: None declared.

## Funding

Deutsche Forschungsgemeinschaft (Fa474/5-1) and Interdisziplinäres Zentrum für Klinische Forschung Münster (core unit PIX).

## Supplementary Material

supplemetary_data_tgab023
