# Supplementary material for: Retrosplenial Cortex Contributes to Network Changes during Seizures in the GAERS Absence Epilepsy Rat Model
Source: Cereb Cortex Commun. 2021 Mar 23;2(2):tgab023. doi: 10.1093/texcom/tgab023 (PMC8263073; doi:10.1093/texcom/tgab023)
Supplement: supplemetary_data_tgab023 [file supplemetary_data_tgab023.pdf]

**Supplementary**

# Retrosplenial cortex contributes to network changes during seizures in the GAERS absence epilepsy rat model

Lydia Wachsmuth[1], Maia Datunashvili[2], Katharina Kemper[1], Franziska Albers[1], Henriette Lambers[1], Annika Lüttjohann[2], Silke Kreitz[3], Thomas Budde[2], Cornelius Faber[1]

[1] University Hospital Münster, Translational Research Imaging Center, Clinic for Radiology, Albert-Schweitzer Campus 1, 48163 Muenster, +49 251 8357608, faberc@uni-muenster.de

[2] University of Münster, Institute of Physiology I

[3] University of Erlangen, Experimental and Clinical Pharmacology and Toxicology

**Running title**

Brain network changes in GAERS

Suppl. Table 1   Animals used for in vivo measurements

| animal ID | # of datasets pre-seizure state | # of datasets seizure state | remarks |
|---|---|---|---|
| GAERS 1 | na | 2 | seizures started early |
| GAERS 2 | na | 1 | seizures started early |
| GAERS 3 | 1 | 2 | |
| GAERS 4 | 1 | 2 | |
| GAERS 5 | 1 | 2 | |
| GAERS 6 | na | 1 | seizures started early |
| GAERS 7 | 2 | 2 | |
| GAERS 8 | na | 2 | seizures started early |
| GAERS 9 | 1 | na | loss of $Ca^{2+}$ signal |
| GAERS 10 | 1 | na | loss of $Ca^{2+}$ signal |
| GAERS 11 | 1 | na | corrupted correlation matrix |
| GAERS 12 | 3 | na | no seizures |
| GAERS 13 | 1 | na | loss of $Ca^{2+}$ signal |
| NEC 1 | 2 | - | |
| NEC 2 | 2 | - | |
| NEC 3 | 2 | - | |
| NEC 4 | 2 | - | |
| NEC 5 | 3 | - | |
| NEC 6 | 3 | - | |
| NEC 7 | 3 | - | |

rs-fMRI was performed with 13 GAERS and 7 NEC. The table lists the number of datasets acquired per animal. In four GAERS seizures started too early to acquire 30 min pre-seizure scans. In three GAERS no Ca-recordings were available in the seizure state, because of excessive bleaching. One GAERS did not show seizures. Correlation matrix of one GAERS was corrupted and was excluded.

Suppl. Table 2 Animals used for immunohistochemistry and slice experiments

| method | strain | n |
|---|---|---|
| immunohistochemical stainings | GAERS | 3 |
| | NEC | 3 |
| voltage clamp recordings | GAERS | 6 |
| | NEC | 7 |
| current clamp recordings | GAERS | 3 |
| | NEC | 3 |

Immunohistochemistry and slice experiments were performed with 12 GAERS and 13 NEC.

Suppl. Table 3   Quality measures

A

| group | | FWD translation/rotation | tSNR | sensitivity above fdr threshold | FC RS-cg |
|---|---|---|---|---|---|
| NEC | left | < 0.2 mm/< 0.3° | 63 ± 8 | 6/7 | 0.459 ± 0.160 |
| | right | | | 6/7 | 0.572 ± 0.115 |
| GAERS pre-seizure | | < 0.2 mm/< 0.3° | 60 ± 6 | - | - |
| GAERS seizure | | < 0.2 mm/< 0.2° | 56 ± 8 | - | - |

B

| | | **specificity** | | | |
|---|---|---|---|---|---|
| group | | above fdr threshold | FC cg-S1HL | above fdr threshold | FC cg-S1r |
| NEC | left | 3/7 | 0.152 ± 0.047 | 2/7 | 0.215 ± 0.146 |
| | right | 0/7 | - | 0/7 | - |

We determined quality measures (A) frame wise displacement (FWD) and temporal signal-to-noise ratio (tSNR). Sensitivity to functional connectivity (FC) was indicated by robust connectivity strength between the cingulate area (cg) and retrosplenial cortex (RS), a central feature of the rodent DMN. Please note that only analysis of the NEC control group theoretically provides an FC-sensitivity measure of general relevance since the pathological model may well influence the connectivity of the DMN. (B) As expected connectivity strengths between the cg and primary sensory cortex (S1HL and S1r) was low. False discovery rate (fdr) threshold was 0.096 ± 0.001 ($p$<0.05).

Suppl. Table 4 Brain regions

| abbreviation | brain region | functional group |
|---|---|---|
| M1 | primary motor cortex | sensorimotor cortex |
| M2 | secondary motor cortex | sensorimotor cortex |
| S1r | primary somatosensory cortex rest | sensorimotor cortex |
| S1HL | primary somatosensory cortex hind limb | sensorimotor cortex |
| S2 | secondary somatosensory cortex | sensorimotor cortex |
| Cg | cingulate cortex | association cortex |
| RS | retrosplenial cortex | association cortex |
| PtA | parietal association cortex | association cortex |
| Ins | insular cortex | association cortex |
| Pir | piriform cortex | link to the limbic system |
| Prh_Ect | perirhinal/ectorhinal cortex | link to the limbic system |
| DB | nuclei of diagonal band | limbic system |
| BST | bed nucleus of stria terminalis | limbic system |
| SLEA | sublenticular extended amygdala | limbic system |
| Amd | amygdala | limbic system |
| Hb | habenuli | limbic system |
| Hip | hippocampus | limbic system |
| Sep | septal area | limbic system |
| Com | corpora mammillaria | limbic system |
| ZI | zona incerta | limbic output |
| HT | hypothalamus | limbic output |
| Rt | reticular thalamic nucleus | thalamus |
| Ant | anterior thalamic group | thalamus/limbic |
| Sub | submedial thalamic nucleus | thalamus |
| MT | medial thalamus | thalamus |
| VM | ventromedial thalamic nucleus | thalamus |
| VA_VL | thalamic nucleus ventralis anterolateralis | thalamus |
| VPL_VPM | ventral postolateral/posteromedial thalamic nucleus | thalamus |
| Po | posterior thalamic nuclear group | thalamus |
| LP | lateral posterior thalamic nucleus | thalamus |
| PV | paraventricular thalamic nucleus | thalamus |
| LG | lateral geniculate nucleus | thalamus |
| VP | ventral pallidum | basalganglia |
| Acb | nucleus accumbens | basalganglia |
| GP | globus pallidus | basalganglia |
| Cpu | striatum | basalganglia |
| Cl | claustrum | basalganglia |

Suppl. Figure 1  Average group connectivity matrices

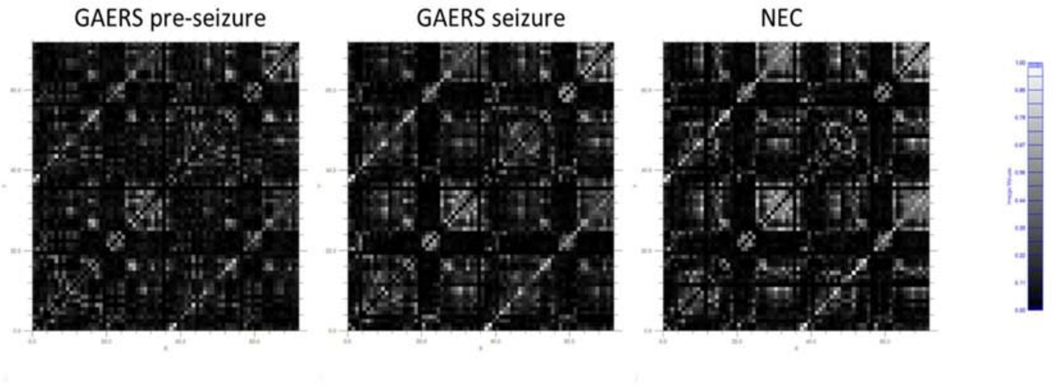


Pearson correlation coefficients of each rs-fMRI-dataset were calculated between pairs of brain regions. This procedure led to a symmetric 72 x 72 cross correlation matrix, representing undirected, weighted functional connections between brain regions. Weights represent respective magnitudes of correlational interactions. Only positive correlation coefficients were considered. If several rs-fMRI-datasets were acquired during one brain state, correlation matrices were averaged per animal. Pearson's r-values were converted to Fisher's z-values to provide normal distribution, averaged, and converted back to Pearson's r-values. The fact that the diagonals, representing the interhemispheric connections between regions are clearly visible indicates that the analysis established meaningful functional connections despite the anatomical distance. GAERS pre-seizure, n = 9, N = 12; GAERS seizure, n = 8, N = 14, NEC, n = 7, N = 17.

Suppl. Figure 2  Local node parameters

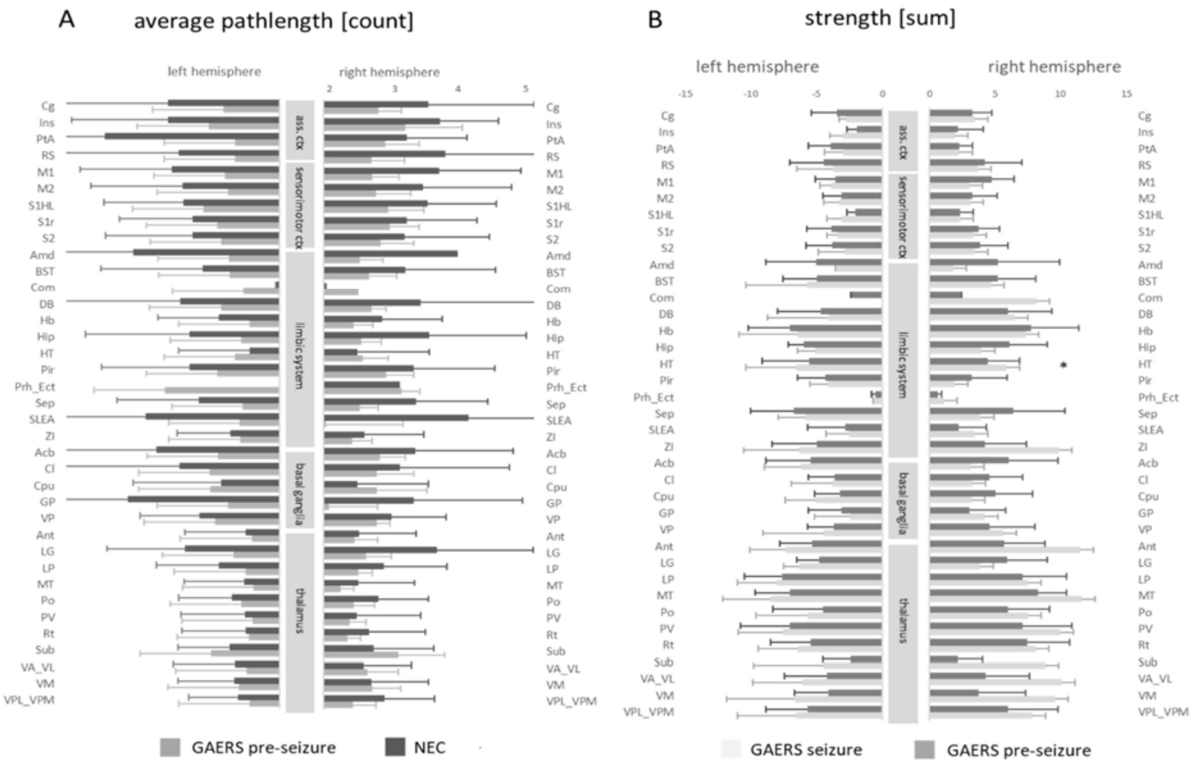


Average local node parameters shortest path length and strength sorted by functional group and hemisphere. (A) Path length did not differ between rat strains. (B) Average local strength of GAERS did not differ between brain states in the vast majority of brain regions, except right hypothalamus. Bars show mean ± SD. Asterisks indicate significant differences, post-hoc t-test, $p < 0.05$, uncorrected.

Suppl. Figure 2 cont.

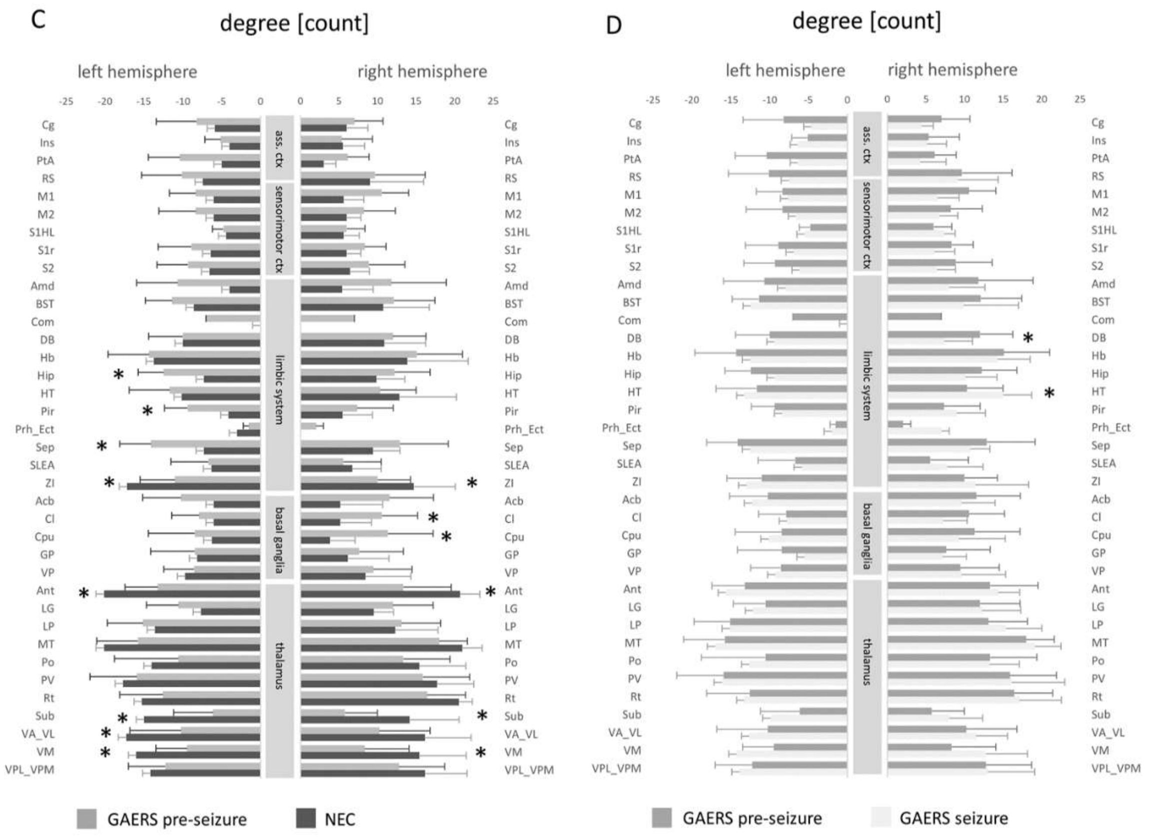


Average local node parameter degree sorted by functional group and hemisphere. (C) Several regions belonging to the limbic system like left hippocampus, septum, and piriform cortex had higher degrees in GAERS pre-seizure when compared to NEC. Thalamic regions anterior nucleus, nucleus ventromedialis, and submedial thalamic nucleus had higher degrees in NEC in both hemispheres. (D) In GAERS, degree did not differ between brain states, except right nucleus of diagonal band (DB) and right hypothalamus. Bars show mean ± SD. Asterisks indicate significant differences, post-hoc t-test, $p < 0.05$, uncorrected.

Suppl. Figure 2 cont.

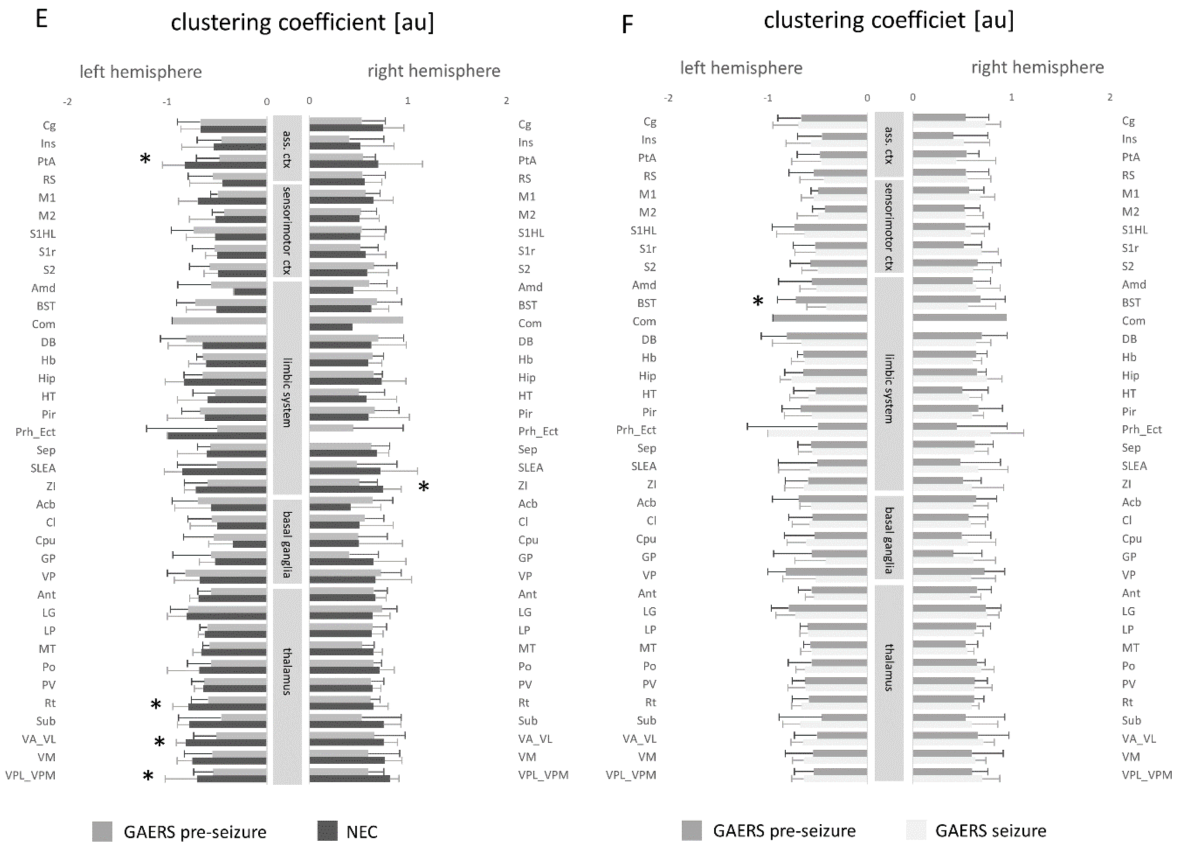


Average local node parameter clustering coefficient sorted by functional group and hemisphere. The vast majority of brain regions showed similar clustering coefficients independent of rat strain and brain state, an agreement with similar global clustering coefficients. Bars show mean ± SD. Asterisks indicate significant differences, post-hoc t-test, $p < 0.05$, uncorrected.

Suppl. Figure 2 cont.

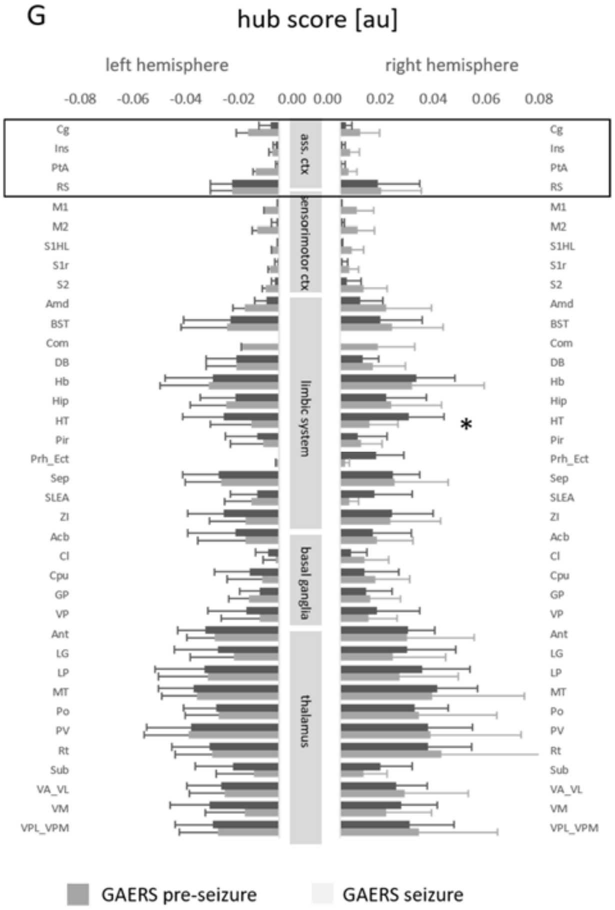


Average local node parameter hub score sorted by functional group and hemisphere. The vast majority of brain regions showed similar hub scores in both brain states. Hub scores were lowest in cortical regions and highest in thalamic regions. Retrosplenial cortex (RS) hub scores were above cortical group average. Regions of association cortex are framed in the box. Bars show mean ± SD. Asterisk indicates significant difference, post-hoc t-test, $p < 0.05$, uncorrected.

Suppl. Table 5A Comparison of local network metrics between brain states

| GAERS pre-seizure vs. GAERS seizure, independent t-test, uncorrected | | | | |
|---|---|---|---|---|
| | | **clustering coefficient** | | |
| brain region | hemisphere | *df* | *t* | *p* |
| BST | left | 13 | -3.083 | **0.009** |
| | | | | |
| | | **shortest pathlength** | | |
| brain region | hemisphere | *df* | *t* | *p* |
| M1 | right | 15 | -3.536 | **0.003** |
| | left | 11 | -3.132 | **0.01** |
| M2 | right | 15 | 3.214 | **0.006** |
| | left | 15 | -3.463 | **0.003** |
| S1r | right | 15 | -2.782 | **0.014** |
| S1HL | right | 15 | -2.489 | **0.025** |
| | left | 15 | -2.551 | **0.022** |
| Cg | right | 15 | -2.691 | **0.006** |
| | left | 15 | -2.641 | **0.017** |
| PtA | right | 15 | 3,414 | **0.007** |
| | left | 14 | 4.247 | **0.001** |
| Ins | right | 15 | -3.414 | **0.007** |
| | left | 14 | -2.17 | **0.048** |
| | | | | |
| | | **degree** | | |
| brain region | hemisphere | *df* | *t* | *p* |
| DB | right | 12 | -2.176 | **0.05** |
| HT | right | 15 | -2.218 | **0.042** |
| | | | | |
| | | **strength** | | |
| brain region | hemisphere | *df* | *t* | *p* |
| HT | right | 15 | 2.627 | **0.019** |
| | | | | |
| | | **hubscore** | | |
| brain region | hemisphere | *df* | *t* | *p* |
| HT | right | 15 | 2.218 | **0.042** |

Suppl. Table 5B Comparison of local network metrics between rat strains

| GAERS pre-seizure vs. NEC, independent t-test, uncorrected | | | | |
|---|---|---|---|---|
| | | clustering coefficient | | |
| brain region | hemissphere | *df* | *t* | *p* |
| PtA | left | 11 | -2.648 | **0.03** |
| ZI | right | 14 | -2.567 | **0.022** |
| Rt | left | 14 | 2.424 | **0.029** |
| VA_VL | left | 14 | -3.3 | **0.005** |
| VPL_VPM | right | 14 | -3.2 | **0.006** |
| | | degree | | |
| brain region | hemisphere | *df* | *t* | *p* |
| Pir | left | 13 | 3.706 | **0.003** |
| Hip | left | 14 | 3.24 | **0.006** |
| Sep | left | 14 | 3.43 | **0.004** |
| ZI | left | 14 | 1.958 | **0.033** |
| Ant | right | 14 | 3.223 | **0.008** |
| Ant | left | 14 | 3.95 | **0.001** |
| Sub | right | 13 | 3.014 | **0.01** |
| Sub | left | 13 | 3.014 | **0.01** |
| VM | right | 14 | 2.385 | **0.032** |
| VM | left | 14 | 9.792 | **0.035** |
| VA_VL | left | 14 | 2.568 | **0.016** |
| Acb | right | 14 | 2.262 | **0.04** |
| Cpu | right | 14 | 3.015 | **0.009** |
| Cl | right | 14 | 2.432 | **0.029** |
| | | strength | | |
| brain region | hemisphere | *df* | *t* | *p* |
| ZI | left | 13 | 2.434 | **0.039** |
| Rt | right | 14 | -2.651 | **0.019** |
| Ant | right | 14 | -3.921 | **0.002** |
| Ant | left | 14 | -4.766 | **<0.001** |
| Sub | right | 7 | -2.751 | **0.028** |
| Sub | left | 8 | -3.007 | **0.018** |
| MT | right | 14 | -3.266 | **0.006** |
| MT | left | 14 | -2.924 | **0.011** |
| VM | right | 14 | -2.505 | **0.039** |
| VM | left | 8 | -2.538 | **0.034** |
| VA_VL | right | 14 | 2.512 | **0.025** |
| VA_VL | left | 14 | -3.331 | **0.005** |
| Cpu | right | 11 | 3.139 | **0.01** |
| | | hubscore | | |
| brain region | hemisphere | *df* | *t* | *p* |
| Ant | right | 14 | 2.901 | **0.009** |
| Ant | left | 14 | 9.625 | **0.014** |
| Sub | right | 14 | -2.222 | **0.043** |
| Sub | left | 13 | -2.915 | **0.03** |
| VA_VL | left | 10 | -2.835 | **0.017** |

Suppl Figure 5B cont.

Obvious differences in local node parameters in GAERS between (A) brain states and (B) rat strains were tested for statistical significance by applying post hoc t-tests. Note that no significant differences in shortest path length between rat strains were detected.  Due to the fact, that consecutive pre-seizure and seizure recordings were only available for three animals (see details in suppl. table 1), we used unpaired t-test. Significant differences are also indicated in the respective figures (Figure 6, 7, and suppl. Figure 2) by asterisk. GAERS pre-seizure, n = 9, N = 12; GAERS seizure, n = 8, N = 14.

Suppl. Table 6 Active and passive membrane properties of Layer 5 pyramidal neurons from NEC and GAERS granular retrosplenial cortex

| | RMP | | | -60 mV | | |
|---|---|---|---|---|---|---|
| Left hemisphere | NEC (n = 10) | GAERS (n = 11) | p | NEC (n = 10) | GAERS (n = 10) | p |
| RMP (mV) | -68.8 ± 1.1 | -66.6 ± 0.9 | 0.2 | -- | -- | -- |
| $R_{in}$ (MΩ) | 118.5 ± 5.4 | 124.1 ± 8.8 | 0.6 | 173.9 ± 12.3 | 169.9 ± 12.1 | 0.8 |
| Cm (pF) | 169.3 ± 10.1 | 183.0 ± 8.7 | 0.3 | 154.8 ± 8.8 | 171.7 ± 8.9 | 0.2 |
| τ (ms) | 19.9± 1.2 | 22.2 ± 1.0 | 0.2 | 26.3 ± 1.3 | 28.5 ± 1.2 | 0.2 |
| Voltage sag amplitude (mV) | 5.8 ± 0.6 | 5.9 ± 0.3 | 0.9 | 9.1 ± 0.8 | 7.8 ± 0.4 | 0.2 |
| Number of rebound spike | --- | --- | --- | 0.9 ± 0.3 | 0.3 ± 0.2 | 0.07 |
| Spike threshold (mV) | -41.2 ± 1.2 | -37.6 ± 0.8 | **0.02** | -41.1 ± 0.8 | -39.4 ± 0.5 | 0.2 |
| Spike amplitude (mV) | 84.9 ± 1.7 | 79.3 ± 2.0 | 0.06 | 84.3 ± 2.4 | 84.9 ± 1.2 | 0.8 |

| | RMP | | | -60 mV | | |
|---|---|---|---|---|---|---|
| Right hemisphere | NEC (n = 22) | GAERS (n = 24) | p | NEC (n = 20) | GAERS (n = 18) | p |
| RMP (mV) | -68.0 ± 0.6 | -67.1 ± 0.8 | 0.4 | -- | -- | -- |
| $R_{in}$ (MΩ) | 113.6 ± 5.7 | 140.6 ± 6.6 | **0.004** | 161.0 ± 6.6 | 195.0 ± 10.3 | **0.005** |
| Cm (pF) | 194.3 ± 9.4 | 182.7 ± 8.9 | 0.4 | 179.9 ± 7.7 | 187.1 ± 9.4 | 0.6 |
| τ (ms) | 21.4 ± 1.0 | 25.4 ± 1.1 | **0.01** | 28.8 ± 1.6 | 36.0 ± 2.0 | **0.008** |
| Voltage sag amplitude (mV) | 4.9 ± 0.3 | 5.9 ± 0.4 | 0.06 | 6.9 ± 0.6 | 6.9 ± 0.5 | 0.9 |
| Number of rebound spike | 0.3 ± 0.2 | --- | 0.2 | 0.4 ± 0.1 | 0.6 ± 0.1 | 0.2 |
| Spike threshold (mV) | -39.8 ± 0.6 | -40.7 ± 0.6 | 0.3 | -39.9 ± 0.5 | -42.9 ± 1.9 | 0.1 |
| Spike amplitude (mV) | 81.9 ± 0.9 | 82.4 ± 1.1 | 0.7 | 83.3 ± 0.9 | 85.2 ± 0.9 | 0.1 |
